# Repairing the Osteochondral Defect in Goat with the Tissue-Engineered Osteochondral Graft Preconstructed in a Double-Chamber Stirring Bioreactor

**DOI:** 10.1155/2014/219203

**Published:** 2014-07-02

**Authors:** Yang Pei, Jun-jun Fan, Xiao-qiang Zhang, Zhi-yong Zhang, Min Yu

**Affiliations:** ^1^School of Public Health, Fourth Military Medical University, No. 17 Changle Road, Xi'an 710032, China; ^2^Department of Orthopaedic Surgery, Xijing Hospital, Fourth Military Medical University, No. 15 Changle Road, Xi'an 710032, China; ^3^Department of Orthopaedic Surgery, Southern Hospital, Southern Medical University, No. 1838 North Guangzhou Road, Guangzhou 510515, China; ^4^Department of Plastic and Reconstructive Surgery, Shanghai 9th People's Hospital, Shanghai Key Laboratory of Tissue Engineering, School of Medicine, Shanghai Jiao Tong University, Shanghai 200040, China

## Abstract

To investigate the reparative efficacy of tissue-engineered osteochondral (TEO) graft for repairing the osteochondral defect in goat, we designed a double-chamber stirring bioreactor to construct the bone and cartilage composites simultaneously in one *β*-TCP scaffold and observed the reparative effect in vivo. The osteochondral defects were created in goats and all the animals were divided into 3 groups randomly. In groups A, the defect was treated with the TEO which was cultured with mechanical stimulation of stir; in group B, the defect was treated with TEO which was cultured without mechanical stimulation of stir; in groups C, the defect was treated without TEO. At 12 weeks and 24 weeks after operation, the reparative effects in different groups were assessed and compared. The results indicated that the reparative effect of the TEO cultured in the bioreactor was better than the control group, and mechanical stimulation of stir could further improve the reparative effect. We provided a feasible and effective method to construct the TEO for treatment of osteochondral defect using autologous BMSCs and the double-chamber bioreactor.

## 1. Introduction

Articular cartilage tissue lacks the blood supply and is difficult to be repaired when damaged [1, 2]. This problem is more severe when the damage involved the subchondral bone. The subchondral bone plays an important role in the stress conduction of the joint and it is also a strong support to make the articular cartilage smooth. Once osteochondral defect occurred in the joint, the abnormal stress distribution and the collapse of articular surface will be inevitable, and a vicious circle will result in more and more damage to the normal articular cartilage [3, 4]. Many studies have been reported for the treatment of osteochondral defect mainly including bone marrow stimulation and cartilage transplantation. However, all the methods mentioned above have their limitations [5, 6].

The development of tissue engineering provides a new way for repairing the osteochondral defects and the complex tissues construction is possible and developed very fast [7–10]. Ideal tissue-engineered osteochondral (TEO) graft should be able to provide better integration of the cartilage and subchondral bone to support better cartilage regeneration. Many studies have already reported on the successful construction of tissue-engineered bone or tissue-engineered cartilage separately [11–13], but the osteochondral complex construction researches are still under the way mainly because of the bad combination of cartilage tissue and subchondral bone tissue [14–16]. To get better integration of the cartilage and subchondral bone, constructing the tissue-engineered osteochondral graft with two kinds of tissue in one scaffold is a hopeful method to avoid the recombining procedure. But how to construct both cartilage and bone tissues in one scaffold is still a big challenge for researchers.

At present, the bioreactor plays a very important role in the development of tissue engineering research and can simulate the in vivo physiological environment of tissues and organs to promote the regeneration [17–21]. We can add the appropriate medium to construct different tissues in vitro. The bioreactor also can provide appropriate mechanical stimulation for the tissue-engineered grafts cultured in the medium and this is very important to construct the tissue-engineered osteochondral graft [22, 23]. For the bone and cartilage tissue, not only physical and chemical factors but also mechanical stimulation plays an important role in the processing of regeneration [24, 25]. Many studies have reported on the successful constructions of bone tissues using the osteogenic differentiation medium cultured in the bioreactor and cartilage tissues using the chondrogenic differentiation medium cultured in the bioreactor [18, 26, 27] But how to use the bioreactor with two kinds of differentiation medium to construct the tissue-engineered osteochondral graft with both the bone and cartilage tissues in one scaffold is still a big question that needs to be solved.

So in this study, we designed a double-chamber stirring bioreactor containing both the osteogenic and chondrogenic differentiation medium, and preconstructed the tissue-engineered osteochondral graft using the goat BMSCs implanted to the *β*-TCP scaffold and cultured in our bioreactor with or without mechanical stimulation of stir in vitro to construct the bone and cartilage composites simultaneously in single scaffold and to observe the reparative effect of the osteochondral defect in goat. We wanted to provide a feasible and effective method to construct the TEO for treatment of osteochondral defect using autologous BMSCs and the double-chamber bioreactor.

## 2. Material and Methods

### 2.1. Isolation, Culture, and Differentiation of Goat BMSCs

**Figure 1:**
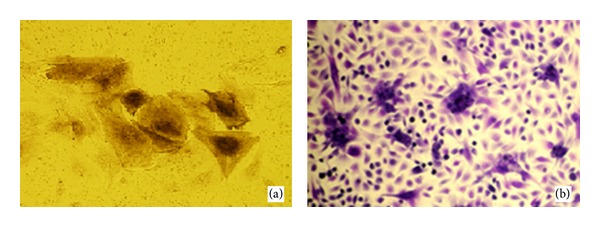
The osteogenic and chondrogenic differentiation of MSCs. (a) The ALP staining of goat BMSCs after osteogenetic induction for 2 weeks (×200). (b) The toluidine blue staining of goat BMSCs after chondrogenic induction for 2 weeks (×40).

All animal experiments were approved by the Institutional Animal Care and Use Committee of Fourth Military Medical University and following the relevant ethical regulations. Number 16 needle was used to aspirate the bone marrow of the goat which was mixed in the non-serum-DMEM-containing heparin (50 U/mL). Lymphocyte separating liquid was added for centrifugation and separation. Then the mononucleated cells were inoculated in the DMEM containing 15% fetal bovine serum (FBS, HyClone, USA) at 37°C in 5% CO_2_ atmosphere. When the cells reached 80–90% confluence of the total area, 2.5 g/l trypsin was used for passage culture. The osteogenic and chondrogenic medium was not added until the third passage. The osteogenic medium consisted of DMEM with 15% FBS, 100 nmol/L dexamethasone, 10 mmol/L *β*-glycerolphosphate, and 50 mg/l Vitamin C. The chondrogenic medium consisted of high glucose DMEM with 15% FBS, 6.25 mg/l insulin, 6.25 mg/l transferrin, 50 mg/l Vitamin C, 100 nmol/L dexamethasone, and 10 ng/mL TGF-*β*1. After 3 weeks of culture, the osteogenic and chondrogenic differentiation of third passage MSCs was confirmed by positive results of alkaline phosphatase and toluidine blue staining (Figures 1(a) and 1(b)).

### 2.2. Manufacture the Double-Chamber Stirring Bioreactor

**Figure 2:**
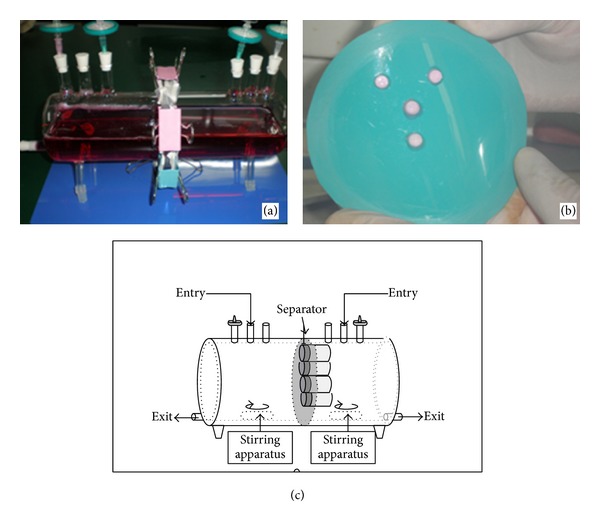
The design of double-chamber stirring bioreactor. (a) The gross observation of double-chamber stirring bioreactor. (b) The separator of double-chamber stirring bioreactor with four scaffolds. (c) The draft of double-chamber stirring bioreactor.

The double-chamber stirring bioreactor was constructed with two glass cylindrical containers integrated compactly and separated into the osteogenic differentiation chamber and chondrogenic differentiation chamber with a separator (Figure 2(a)). In the middle of the separator, there are four channels with a diameter of 6 mm just as the diameter size of the *β*-TCP scaffold (Figure 2(b)). The differentiation medium was added into the respective chamber through the entry holes. There was an air vein for each chamber to change the air and an exit hole at the base of the chamber to change the medium. The bioreactor was placed on the top of magnetic stirring apparatus and there was one magnetic stirring bar in each chamber to stir the medium at a speed of 300 rpm and provide the mechanical stimulation (Figure 2(c)).

### 2.3. The Preconstruction of Tissue-Engineered Osteochondral Graft

**Figure 3:**
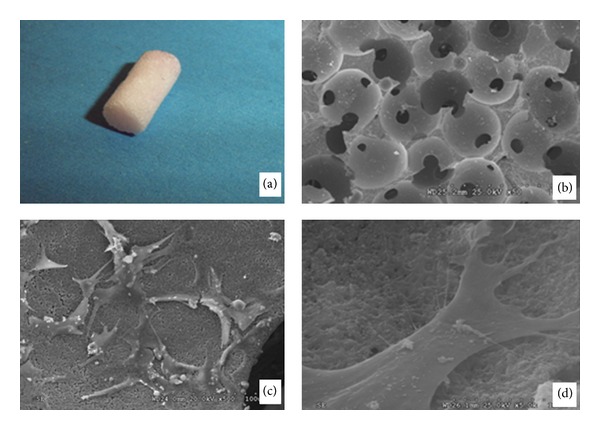
*β*-TCP composite scaffold with goat BMSCs. (a) General observation of the *β*-TCP cylinder scaffold (diameter: 6 mm; length: 12 mm). (b) The porous internal structure of the scaffold under SEM (×50). (c) Observation of BMSCs attached on the surface of the materials under SEM (×500). (d) Observation of BMSCs attached on the surface of the materials under SEM (×5000).

The cylinder scaffold (diameter: 6 mm; length: 12 mm) was custom-made by Mathys Company (Shang Hai, China) with *β*-TCP (Figure 3(a)). The scaffold was porous with fine intensity and packaged sterilely (porosity: 60 ± 10%, spherical pores: 130 ± 20 *μ*m in diameter) (Figure 3(b)). Before being utilized, the scaffold had been conditioned with DMEM for 1 h before cell loading. The autologous MSCs were loaded onto *β*-TCP scaffold to get the autologous TEO graft and cultured over night for cell adhesion before culture in the bioreactor (Figures 3(c) and 3(d)). Then four TEO scaffolds were inserted into four channels of the separator with about 2 mm length in the chondrogenic differentiation chamber and 10 mm length in the osteogenic differentiation chamber. The gaps between the scaffold and the separator were sealed with a kind of gel made with sodium alginate and calcium chloride to avoid the medium leaking between two chambers. The medium was added to soak the scaffolds. After 2 weeks of culturing in the bioreactor, the tissue-engineered osteochondral graft was harvest and implanted into the osteochondral defect of the goat.

### 2.4. Animal and Surgery Procedures

**Figure 4:**
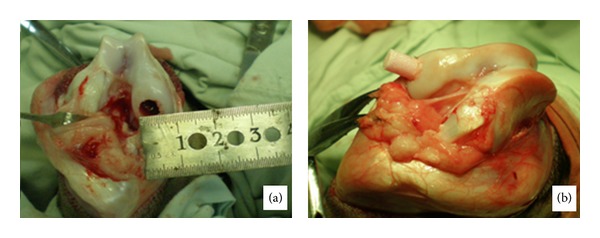
The goat osteochondral defect and the implantation of BMSCs-*β*-TCP scaffold. (a) An osteochondral defect of 6 mm in diameter and 12 mm in depth was created in the femoral medial condyle weight-bearing areas of posterior limbs. (b) The implantation of BMSCs-*β*-TCP composite.

For each goat, an osteochondral defect of 6 mm in diameter and 12 mm in depth was created in the femoral medial condyle weight-bearing areas of both two posterior limbs. Drilling at weight-bearing cartilage with the external diameter of 6 mm trepan, bone and cartilage debris was removed (Figure 4(a)). All 12 goats were divided into three groups: group A: TEO was cultured in double-chamber bioreactor with mechanical stimulation of stirring before implantation; group B: TEO was implanted without being cultured in double-chamber bioreactor and mechanical stimulation; groups C: the defect was treated without TEO. Each group had 4 goats and 8 posterior limbs. In group A and group B, autologous TEO graft preconstructed previously or TEO only was implanted into the osteochondral defect area (Figure 4(b)). After the implantation, the gel formed by sodium alginate and calcium chloride was covered on the surface of defect. In group C, there is nothing implanted in the defect site. The goats in each group were sacrificed at 12 weeks and 24 weeks after operation. Then the reparative effect of the osteochondral defect in each group was assessed by the general observation, HE staining, toluidine blue staining, Masson staining, collagen II immunohistochemistry, and O'Driscoll score.

### 2.5. Histological and Immunohistochemical Evaluation

Two goats in all groups were anesthetized and the samples of the TEO graft were taken at 12 and 24 weeks after the operation. Eight samples from each group were used for histological and immunohistological assessment. All the samples were fixed in 4% buffered paraformaldehyde, decalcified in 50 mM ethylenediaminetetraacetic acid (EDTA), embedded in paraffin, and sectioned at 5 mm thickness. The sections were prepared sagittally and stained with hematoxylin and eosin (HE), Masson's trichrome staining, and toluidine blue staining according the standard method. Immunohistochemistry was performed with rabbit antibodies for type II collagen. Sections were treated with 0.5% pepsin in 5 mM HCl at 37°C for 30 min for epitope unmasking. After overnight incubation at 4°C with a rabbit anti-type II collagen polyclonal antibody (1 : 2000), sections were incubated with an anti-rabbit secondary antibody (1 : 200) for 30 min at room temperature. A Vectastain ABC kit and DAB substrate system were used for color development.

O'Driscoll histomorphology score in each group was assessed independently by three independent experienced examiners in a blinded manner and compared among different groups according to the criterion system, which was frequently used for cartilage analysis in animal studies and suitable for analysis of in vivo repaired cartilage [28].

### 2.6. Statistical Analysis

All data were analyzed using SPSS software. The data were expressed as mean ± standard deviation (SD) and levels were compared by a one-way analysis of variance and Student's *t*-test. *P* values less than 0.05 were considered significant.

## 3. Results

### 3.1. Gross Observation

**Figure 5:**
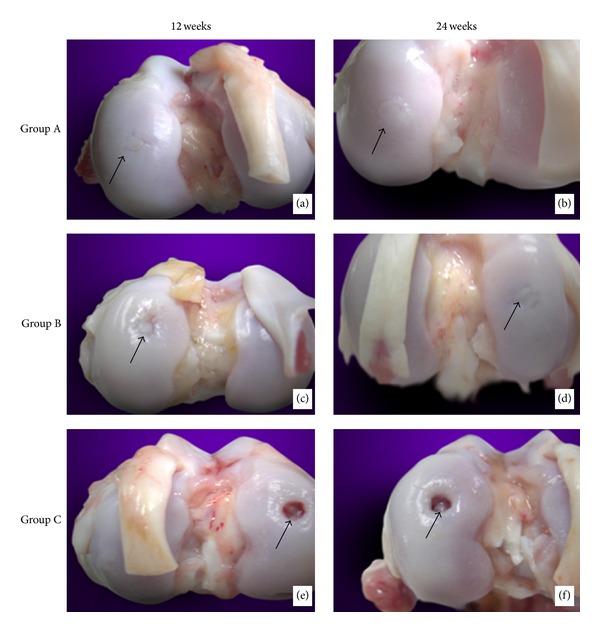
General observation of the osteochondral area after operation. (a) 12 weeks after operation of group A. (b) 24 weeks after operation of group A. (c) 12 weeks after operation of group B. (d) 24 weeks after operation of group B. (e) 12 weeks after operation of group C. (f) 24 weeks after operation of group C.

After 12 weeks, the result showed the articular surface in group A was a little unsmooth with a lacuna. The defect site was covered with the semitransparent tissues like the normal cartilage tissue and connected with the normal cartilage tissue (Figure 5(a)); in group B, the articular surface was more unsmooth than group A with an obvious lacuna. The defect site was covered with little semitransparent tissues like the normal cartilage tissue and disconnected with the normal cartilage tissue; the subchondral bone tissue was not obviously exposed (Figure 5(c)). In group C, there was no articular cartilage tissue in the defect site and the subchondral bone tissue was obviously exposed (Figure 5(e)); after 24 weeks, the articular area in group A was very smooth. The defect site was covered with the semitransparent tissues like the normal cartilage tissue and connected with the normal cartilage tissue (Figure 5(b)). In Group B, the articular surface was smoother than before but still has a lacuna. The defect site was covered with more semitransparent tissues like the normal cartilage tissue and connected with the normal cartilage tissue; the subchondral bone tissue was not obviously exposed (Figure 5(d)). In group C, there was still no articular cartilage tissue in the defect site and the subchondral bone tissue was still obviously exposed just like before (Figure 5(f)).

### 3.2. Histological Evaluation

**Figure 6:**
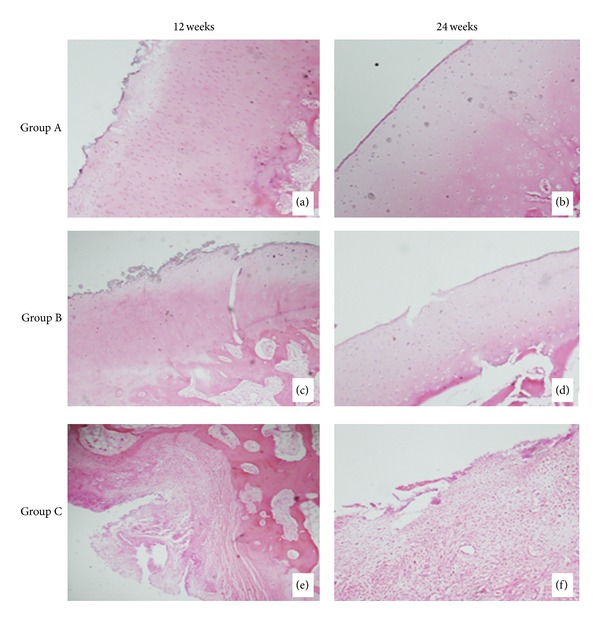
HE staining of groups A, B, and C at 12 weeks and 24 weeks postoperatively (×40). (a) HE staining of group A at 12 weeks. (b) HE staining of group A at 24 weeks. (c) HE staining of group B at 12 weeks. (d) HE staining of group B at 24 weeks. (e) HE staining of group C at 12 weeks. (f) HE staining of group C at 12 weeks.

**Figure 7:**
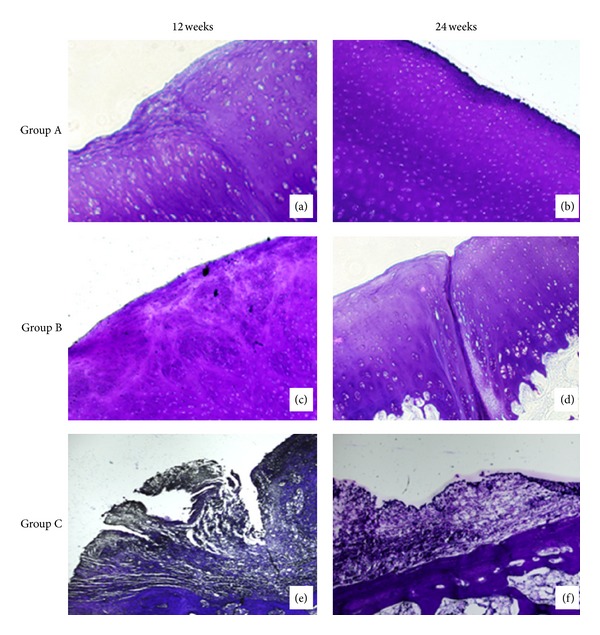
Toluidine blue staining of groups A, B, and C at 12 weeks and 24 weeks postoperatively (×40). (a) Toluidine blue staining of group A at 12 weeks. (b) Toluidine blue staining of group A at 24 weeks. (c) Toluidine blue staining of group B at 12 weeks. (d) Toluidine blue staining of group B at 24 weeks. (e) Toluidine blue staining of group C at 12 weeks. (f) Toluidine blue staining of group C at 12 weeks.

**Figure 8:**
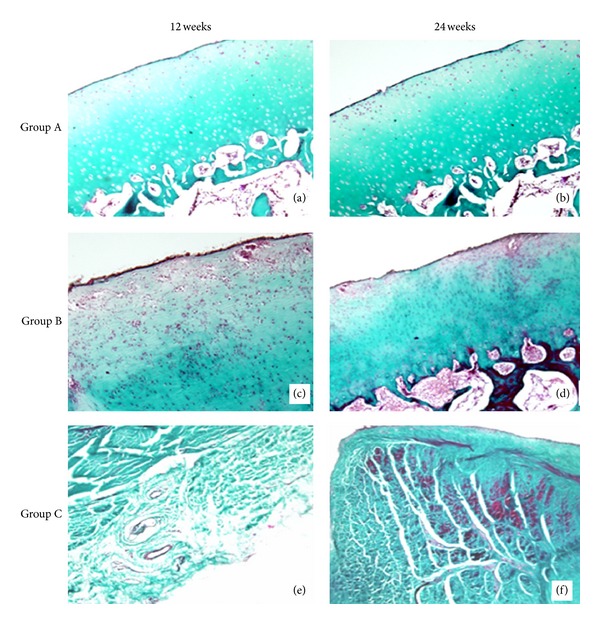
Masson staining of groups A, B, and C at 12 weeks and 24 weeks postoperatively (×40). (a) Masson staining of group A at 12 weeks. (b) Masson staining of group A at 24 weeks. (c) Masson staining of group B at 12 weeks. (d) Masson staining of group B at 24 weeks. (e) Masson staining of group C at 12 weeks. (f) Masson staining of group C at 12 weeks.

At 12 weeks, HE staining result showed that the new cartilage tissue with the mature structure of cartilage lacuna and the subchondral bone tissues appeared in groups A and B, but only fibrous tissue was shown in group C and the surface of the cartilage in all groups was rough. Group A had the best cartilage tissues appearance, Figures 6(a), 6(c), and 6(e). At 24 weeks, there was still only fibrous tissue in group C and more cartilage tissues were shown in groups A and B. The surface of the cartilage was very smooth in group A and a little rough in group B. Group A had the best reparative effect, Figures 6(b), 6(d), and 6(f). The results of Masson's trichrome staining and toluidine blue staining were just similar to the HE staining in all the groups, Figures 7 and 8. At 12 weeks, Masson's trichrome staining and toluidine blue staining showed that the chondrocytes were stained in group A and group B but nothing in group C. And the structure of the cartilage matrix was the best in group A and there were only fibrous tissues shown in group C. At 24 weeks, Masson's trichrome staining and toluidine blue staining showed that more chondrocytes were stained in group A and group B but still nothing in group C. And the structure of the cartilage matrix was best in group A with a smooth surface and neatly arranged matrix. There was still no chondrocyte or cartilage matrix shown in group C. Nevertheless, much more pronounced cartilage regeneration was shown in group A compared with the other two groups, and there was no cartilage tissue shown in group C at both 12 and 24 weeks.

### 3.3. Immunohistochemistry Evaluation

**Figure 9:**
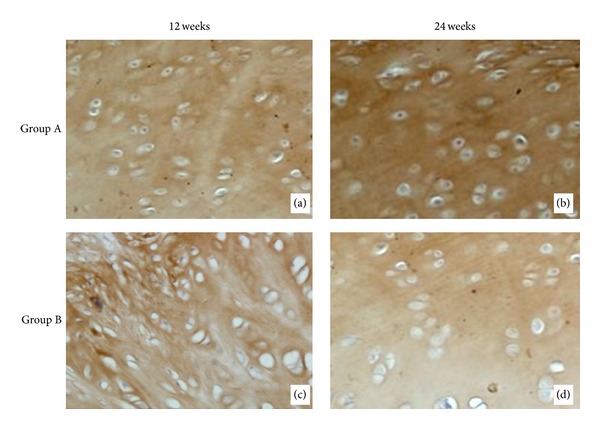
The immunohistochemistry staining of collagen II in groups A and B at 12 weeks and 24 weeks postoperatively (×200). (a) The immunohistochemistry staining of collagen II in group A at 12 weeks. (b) The immunohistochemistry staining of collagen II in group A at 24 weeks. (c) The immunohistochemistry staining of collagen II in group B at 12 weeks. (d) The immunohistochemistry staining of collagen II in group B at 24 weeks.

Collagen II of the cartilage tissues in group A and group B at 12 and 24 weeks was shown as the brown staining area (Figure 9). Because there was no formation of cartilage tissue in group C, we did not take the samples to collagen II immunohistochemistry study. At 12 weeks, the repair tissue was strongly stained for type II collagen indicating a mature hyaline-like cartilage repair tissue had been produced (Figure 9(a)). In group B, there was also type II collagen stained but the structure was disordered (Figure 9(c)). At 24 weeks, more intense staining of type II collagen was shown and the structure was good (Figure 9(b)). In group B, there was also type II collagen stained and the structure was better than before (Figure 9(d)).

### 3.4. O'Driscoll Histomorphology Scores

**Figure 10:**
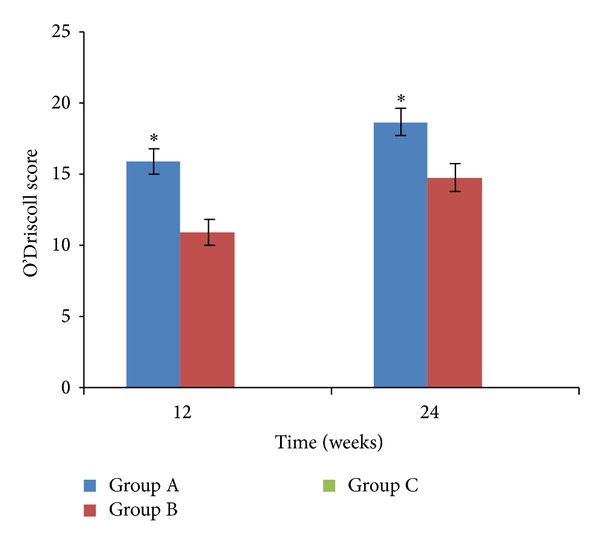
The O'Driscoll scores of the histomorphology in group A and group B at 12 weeks and 24 weeks postoperatively (*P < 0.05 versus group B).

The O'Driscoll histomorphology scores of different groups at 12 and 24 weeks were shown in Figure 10. Statistically significant differences were found at 12 and 24 weeks between group A and group B. At different time points, the scores in group A were higher than group B (*P < 0.05). With the time increasing, the scores were significantly increased both in group A and in group B. In group C at 12 and 24 weeks, there was no cartilage tissue and the score was zero.

## 4. Discussion

The osteochondral defects were difficult for clinical surgeons to be repaired using the traditional methods. The tissue engineering provided a new way for repairing the osteochondral defects and the complex tissues construction is possible and developed very fast [7–10]. Ideal tissue-engineered osteochondral (TEO) graft should be able to provide a better integration of the cartilage and subchondral bone to support better cartilage regeneration. And the bioreactor played a very important role in the culture of TEO graft in vivo. Many researches have reported on the successful constructions of bone tissues using the osteogenic differentiation medium cultured in the bioreactor and cartilage tissues using the chondrogenic differentiation medium cultured in the bioreactor [18, 26, 27]. But how to use the bioreactor with two kinds of differentiation medium to construct the tissue-engineered osteochondral graft with both the bone and cartilage tissues in one scaffold is still a big question that needs to be solved. In our study, we designed a double-chamber stirring bioreactor containing both the osteogenic and chondrogenic differentiation medium to preconstruct the tissue-engineered osteochondral graft in vitro. We combined two chambers together and used a separator between two chambers to separate them. When the scaffold was inserted into the channels of the separator and the gaps between the scaffold and the separator were sealed with a kind of gel made with sodium alginate and calcium chloride, we could avoid the leaking of the medium between two chambers. And our design of the double-chamber bioreactor made the construction of the tissue-engineered osteochondral graft in one scaffold possible. In our study, we successfully repaired the osteochondral defect using this scaffold cultured in our bioreactor and found that the scaffold cultured in our double-chamber bioreactor could form both the bone tissues and the cartilage tissues simultaneously in vivo.

And the mechanical factors influence the biological behavior of chondrocytes in an extremely complex process involving cell morphology, growth, differentiation, and distribution. Research has proved that external forces can affect the BMSCs differentiation towards osteoblasts and chondrocytes, which suggest that mechanical factors may have multilineage differentiation potential for stem cells [25]. Different mechanical forces in different strength and frequency can adjust and maintain normal chondrocyte biology through a mechanical signal transduction mechanism. Studies have found that stress load can adjust chondrocyte proliferation and matrix metabolism in vitro [18]. Although this mechanism of mechanical stimulation has not yet been fully clarified, it suggests that chondrocytes cultured in vitro with a certain dynamic mechanical stimulation could promote the secretion of extracellular matrix such as the proteoglycan and collagen [27]. In our study, we found that the scaffold cultured in our bioreactor with a mechanical stimulation of stir at a speed of 300 rpm had a better reparative effect than the scaffold cultured without the stir. But the optimal mechanical stimulation was still unknown and needed to be further studied.

Calcium phosphate bioceramics have been widely accepted as an excellent scaffold for bone tissue engineering [29, 30]. *β*-TCP had good biocompatibility, degradability, and bone conduction capacity to be used in the tissue-engineered bone and got a splendid repair effect [31, 32]. *β*-TCP also has been used to construct tissue-engineered cartilage and researchers have successfully used the *β*-TCP as the scaffold to construct cartilage tissue with chondrocyte and stem cells [33, 34]. So we used the *β*-TCP scaffold to construct the tissue-engineered osteochondral graft including both the bone and cartilage tissues in our bioreactor. Our results showed that the *β*-TCP scaffold cultured in our bioreactor could form both the bone tissues and the cartilage tissues simultaneously in vivo and the reparative effect was the best in the group with the TEO cultured in the bioreactor combined with the mechanical stimulation. So we may provide a feasible and effective method to construct the TEO for treatment of osteochondral defect using autologous BMSCs and the double-chamber bioreactor.

## 5. Conclusions

We designed a double-chamber stirring bioreactor containing both the osteogenic and chondrogenic differentiation medium to preconstruct the tissue-engineered osteochondral graft with or without mechanical stimulation of stir in vitro. And we used the preconstruct tissue-engineered osteochondral graft including both the bone and cartilage tissues to repair the osteochondral defect in goat and observed the reparative effect in vivo. The results indicated that the reparative effect of the TEO cultured in the bioreactor was better than the blank group, and mechanical stimulation of stir could further improve the reparative effect. So we may provide a feasible and effective method to construct the TEO for treatment of osteochondral defect using autologous BMSCs.
